# Comparison of Short-Term Results between Patients Undergoing Coronary Artery Bypass Graft with a Stent-Placement History and Patients Undergoing Primary Coronary Artery Surgery

**Published:** 2019-04

**Authors:** Mehdi Hadadzadeh, Mahdiesadat Mirahmadi, Masoud Mirzaei, Ali Pedarzadeh, Bahram Ghasemzadeh, Ali Akbar Rahimianfar

**Affiliations:** 1 *Yazd Cardiovascular Research Center, Shahid Sadoughi University of Medical Sciences, Yazd, Iran.*; 2 *Department of Cardiac Surgery, School of Medicine, Shiraz University of Medical Sciences, Shiraz, Iran.*

**Keywords:** *Coronary artery disease*, *Angioplasty, balloon, coronary*, *Percutaneous coronary intervention*, *Coronary artery bypass*

## Abstract

**Background**: Percutaneous coronary intervention (PCI) has become the first-choice treatment strategy the world over for patients with chronic coronary artery disorders. This study compared the effects of previous PCI procedures on the short-term postoperative results of coronary artery bypass graft surgery (CABG).

**Methodsː** This cross-sectional analytical study recruited 220 patients who underwent CABG in Afshar Hospital in the Iranian city of Yazd between March 2009 and February 2013. The mean postoperative morbidity and mortality rates, the mean postoperative left ventricular ejection fraction (LVEF), the mean hemorrhage volume, the mean serum urea level, and the mean length of stay in the intensive care unit (ICU) were compared between the PCI and non-PCI groups.

**Results:** Among the 220 participants, 147(66.8%) were male and 73(33.2%) were female. The mean age of the study population was 59.41±10.52 years. There was no significant difference in the risk of mortality between the 2 groups (P=0.369). The mean serum urea level was 21.14±6.52 mg/dL in the PCI group and 14.45±1.08 mg/dL in the non-PCI group (P=0.016). The mean postoperative LVEF was 43.19±8.81% in the PCI group and 45.51±8.15% in the non-PCI group (P=0.044). The mean length of stay in the ICU was 3.34±1.23 days in the PCI group and 2.22±0.56 days in the non-PCI group (P<0.001). The mean hemorrhage volume was 1113.01±428.13 mL in the PCI group and 961.42±228.31 mL in the non-PCI group (P=0.027).

**Conclusion:** Previous PCI procedures did not affect the post-CABG mortality rate; however, some postoperative results were worse in the PCI group than in the non-PCI group, which should be considered before the selection of the revascularization method.

## Introduction

The prevalence of coronary artery disease has increased in several developing countries in Central Asia and the Middle East, with many of these patients needing interventional treatments.^        1 ^ When the patient is qualified for both percutaneous coronary intervention (PCI) and coronary artery bypass graft surgery (CABG), PCI is usually preferred. Many experts believe that in the case of PCI failure, the patient may be referred for CABG without surgical complications.^2^, ^3^ In fact, the myocardial revascularization model has changed considerably over the last 2 decades4 and the use of PCI as the primary and principal treatment strategy has been well accepted.^2^, ^5^, ^6^ Despite advancements in the PCI technology, the negative outcomes of PCI have increased significantly,^   6 ^ including recurrent stenoses and repeated signs of ischemia.^   7 ^ Previous investigations have reported rates of between 14% and 30% for post-stenting revascularization: 78% repeated PCI and 22% CABG.^5^, ^8^-^10^ Other studies have demonstrated that between 10% and 30% of patients undergo CABG following PCI due to the failure of PCI and recurrent stenoses.^2^, ^6^, ^11^-^13^ The proved local systemic inflammatory response after PCI may affect the results after CABG.^   14 ^ There are no clear-cut confirmed results as to whether PCI can serve as an independent risk factor influencing the negative results after CABG. The present study aimed to compare short-term outcomes, including morbidity and mortality, between CABG patients with a past history of PCI following CABG and patients undergoing primary CABG straight away.

## Methods

This cross-sectional analytical study was conducted on the patients who underwent off-pump CABG in Afshar Hospital in Yazd, Iran, between March 2009 and February 2011. The patients were divided into PCI and non-PCI groups. All the patients with previous PCI procedures undergoing CABG during this period were included (n=143). After the exclusion of the patients who failed to meet the inclusion criteria, 110 patients were recruited and were compared with the same number of patients with no PCI before CABG.

The study protocol was approved by the Ethics Committee of Shahid Sadoughi University of Medical Sciences, and informed written consent was obtained from all the participants prior to inclusion in the study.

Patients who had third-generation stents and no history of previous CABG or other major cardiac interventions were included in the PCI group, while those without PCI and without a history of previous CABG were included in the non-PCI group. Furthermore, patients who needed other interventions such as valve and aneurysm repair along with CABG, patients who needed emergency CABG, and patients who underwent on-pump surgery were excluded from both groups.

All the patients underwent radical myocardial revascularization using off-pump CABG. The anesthesia method was the same for both groups. Aspirin taken by the patients was discontented on the day of surgery and resumed on the second postoperative day. Clopidogrel was discontinued 2 to 5 days before the day of the procedure and resumed 2 days after surgery. The patients were wheeled into the intensive care unit (ICU) after surgery and monitored for 48 hours. They were visited daily by cardiologists. Complete blood count, electrolyte tests, and renal tests were carried out on a daily basis. Echocardiography was done for the patients, and their ventricular function and valvular disturbances were recorded.

The data were collected in a checklist using hospital records. The researcher recorded the data related to both groups including demographic features, a history of vascular and non-vascular diseases, coronary risk factors, the number and pattern of the involvement of the epicardial coronary arteries, the number of grafted arteries, the length of stay in the hospital and the ICU, the anti-arrhythmic drugs taken, renal failure, cerebrovascular accidents, myocardial infarction, the left ventricular ejection fraction (LVEF), the hemorrhage volume, and the postoperative mortality rate. The data for the patients with a previous PCI procedure, including the number of arteries undergoing PCI and the time interval between PCI and CABG, were also recorded. Additionally, at the time of discharge, each patient’s data were collected and recorded in the checklist. The SPSS software, version 21.0 (Armonk, NY: IBM Corp.) was used to analyze the data. The patients’ age and the time interval between PCI and CABG were reported as mean±standard deviations (SDs). The categorical data were analyzed using the χ2 and the Fisher exact test. These categorical data comprised demographic features, cardiac and noncardiac diseases, coronary risk factors, the involvement of the left main artery, valvular involvement, aneurysms, and the drugs consumed before and after surgery. Age, the LVEF before and after surgery, the rate of bleeding, the rate of fresh frozen plasma, the rate of packed cells, the level of creatinine, the level of hemoglobin and the rate of urea rate after surgery, and the length of stay in the ICU and the hospital were normally distributed in the 2 study groups and were reported as mean±SDs and compared using the t-test. The findings were considered statistically significant if a P value was less than 0.05.

## Results

Among the 220 patients enrolled in the study, 147 (66.8%) were male and 73 (33.2%) were female. The mean age of the patients was 59.41±10.52 years. In 84.5% of the patients in the PCI group, stents were placed in the left anterior descending artery, in 19.1% in the left circumflex artery, and in 14.5% in the right coronary artery. Stents were placed in 2 arteries for 21 patients. The PCI procedures were performed from 1 month to 10 years (mean±SD=3.34±1.21 y) before CABG. There was no significant difference in the mortality rate between the 2 groups. Apropos of postoperative mortality, there were 4 cases in the PCI group and 1 case in the non-PCI group (P=0.369).

There were no statistically significant differences between the demographic features, a history of cardiac and noncardiac diseases, coronary risk factors, the involvement of the left main artery, valvular involvement, and the LVEF before surgery between the 2 groups. A history of valvular involvement and aneurysms were not found in any of the patients before CABG. The patients in both groups were similar regarding the drugs taken preoperatively, including beta-receptor antagonists and calcium-channel blockers; however, they were significantly different with respect to the consumption of statins (49 [44.5%] in the PCI group vs 20 [18.2%] in the non-PCI group; P<0.001) (Table 1).

The postoperative outcomes are summarized in Table 2. One cerebrovascular accident occurred in the non-PCI group. Two cases of renal failure were reported in the group with a previous PCI procedure. There were 2 cases of infection in each group. Myocardial infarction was not reported in either group. Two patients in the PCI group developed postoperative valvular disorders. Twenty-seven patients in the PCI group and 23 patients in the non-PCI group developed postoperative arrhythmias. Twenty-one patients in the PCI group and 15 patients in the non-PCI group needed inotropes. The need for amiodarone was reported in 6 patients in the PCI group and 4 patients in the non-PCI group.

**Table 1:** Comparisons of the preoperative characteristics between the PCI and non-PCI groups*

	PCI Group (n=110)	Non-PCI Group (n=110)	P
Age (y)	59.41±10.52	59.6±10.2	0.594
Sex			
Male	70 (63.6)	77 (70.0)	0.316
Female	40 (36.4)	33 (30.0)	
COPD	3 (2.7)	4 (3.6)	0.996
Addiction	6 (5.5)	10 (9.1)	0.299
Left main>50%	15 (13.6)	21 (19.1)	0.274
Hypertension	48 (43.6)	47 (42.7)	0.892
Diabetes mellitus	51 (46.4)	44 (40.0)	0.341
Cerebrovascular accident	2 (1.8)	2 (1.8)	0.998
Renal failure	1 (0.9)	4 (3.6)	0.369
Unstable angina	31 (28.2)	21 (19.1)	0.113
Myocardial infarction	36 (32.7)	24 (21.8)	0.069
Smoking	31 (28.2)	39 (35.5)	0.247
LVEF (%)	48.02±9.81	49.02±9.81	0.417
Atorvastatin	49 (44.5)	49 (44.5)	<0.001
Β-blockers	77 (70.0)	64 (58.2)	0.068
CCB	16 (14.5)	17 (15.5)	0.085

PCI, Percutaneous coronary intervention; COPD, Chronic obstructive pulmonary disease; LVEF, Left ventricular ejection fraction, CCB, Calcium-channel blocker

* Data are presented as mean±SD or n (%).

**Table 2:** Comparisons of the postoperative characteristics between the PCI and non-PCI groups*

Outcome	PCI Group (n=110)	Non-PCI Group (n=110)	P
Cerebrovascular accident	0	1 (0.9)	0.997
Renal failure	2 (1.8)	0	0.496
Infection	2 (1.8)	2 (1.8)	0.998
Inotrope	21 (19.1)	15 (13.6)	0.274
Arrhythmia	27 (24.50)	23 (20.9)	0.526
Amiodarone	6 (5.5)	4 (3.6)	0.748
Valvular disease	2 (1.8)	0	0.498
Urea (mg/dL)	21.14±6.52	14.45±1.08	0.016
Creatinine (mg/)	1.15±0.28	1.12±0.25	0.173
Hemoglobin (g/dL)	13.11±3.71	14.42±2.65	0.085
LVEF (%)	43.19±8.82	45.51±8.15	0.044
ICU stay (d)	3.34±1.23	2.22±0.56	<0.001
Hospital stay (d)	6.46±2.74	5.53±1.12	<0.001
Bleeding (mL )	1113.01±428.13	961.42±228.31	0.027
Packed cells (unit)	2.62±0.81	2.01±0.42	0.163
FFP (unit)	3.27±34.31	0	0.756

PCI, Percutaneous coronary intervention; MI, Myocardial infarction; LVEF, Left ventricular ejection fraction; FFP, Fresh frozen plasma

* Data are presented as mean±SD or n (%).

 The mean length of stay in the ICU was 3.34±1.23 days for the PCI group and 2.22±0.56 days for the non-PCI group (P<0.001). The mean of the hospital length of stay was 6.46±2.74 days for the PCI group and 5.53±1.12 days for the non-PCI group (P<0.001). The postoperative LVEF was 43.19±8.81% in the PCI group and 45.51±8.15% in the non-PCI group (P=0.044). There was a significant difference in terms of the urea rate between the 2 groups after CABG (P=0.016): the urea rate was 21.14±6.52 mg/dL in the PCI group and 14.45±1.08 mg/dL in the non-PCI group. There were no significant differences between the 2 groups as regards the creatinine and hemoglobin levels postoperatively. Furthermore, there was a significant difference in the bleeding rate between the PCI and non-PCI groups (1113.01±428.13 mL vs. 961.42±228.31 mL; P=0.027). The rates of fresh frozen plasma and packed cells were the same in both groups.

## Discussion

The present study demonstrated that the PCI and non-PCI groups were similar regarding postoperative mortality, but there were significant differences between the 2 groups with respect to some morbidities after CABG. The mean bleeding rate, the mean postoperative LVEF, the mean urea rate, the mean ICU length of stay, and the mean hospital length of stay were significantly different between the groups.

Contrary to our findings, Lisboa et al.^   4 ^ reported that the mortality rate was higher in their PCI group than in their non-PCI group. However, the authors used on-pump CABG, while we applied off-pump CABG. Several studies have revealed that cardiopulmonary bypass induces an inflammatory response, affecting post-CABG morbidity.^15^, ^16^ The difference in the method may have contributed to the discrepancy between the reported results. Additionally, all the study samples of Lisboa and colleagues had the involvement of multiple arteries and there was no exclusion of the patients in need of emergency CABG, while we sought to reduce the probable effects of these factors on the outcomes by excluding patients who needed emergency CABG. 

A study conducted by Toshihiro Fukui et al.^   14 ^ showed no postoperative difference in the mortality rate of CABG patients, which was similar to our study. Nevertheless, in contrast to our findings, there was no difference between the 2 groups regarding the mean postoperative hemorrhage volume and the other postoperative results. We tried to decrease the probable effects of these risk factors on the outcomes by expanding the exclusion criteria and recording the similarity between the other risk factors. In addition, in the study by Fukui and colleagues, more patients in the non-PCI group were female, and the fact that female patients are liable to sustain higher risks of morbidity and mortality following CABG than males may explain the difference. In addition, in that study, the number of involved arteries in the patients without a PCI history was greater than that in ours. Their exclusion criteria were also more limited than ours, which might have affected the outcomes of the study. 

In discordance with the results of our study, in a study by Eifert et al.,^   17 ^ the postoperative mortality rate was significantly different between the 2 groups and was greater in the PCI group. In that study, the number of patients with 2 stents was more than that in our study. In addition, 6 patients had 3 stents, while none of our patients had 3 stents, indicating that patients with a previous PCI procedure were at a higher risk than were our participants. The authors reported that all their patients underwent on-pump CABG. Further, not only did the patients with a previous PCI procedure show more comorbidities and more severe coronary artery diseases than did the other group, but also a greater number of the patients with a previous PCI procedure underwent emergency CABG and the follow-up time of the study was longer than ours. Unlike our study, in which the hemorrhage volume in the patients with a previous PCI procedure was greater than that of the other group, the bleeding rate in the patients without a previous PCI procedure was greater than that in the patients with a previous PCI procedure in the study by Eifert and colleagues. This result was obtained while more concentrated (packed) platelets were used for the patients with a previous PCI procedure, and the difference in the bleeding rate between the 2 groups may be attributable to the dose of thrombocytes used. 

Although the distribution of mortality after CABG was not statistically different between our 2 study groups, the mortality rate was greater in the PCI group than in the non-PCI group (3.6% vs. 0.9%). In our study, the transfusion of blood products, the use of amiodarone, postoperative arrhythmias, valve involvement, the need for inotropes, and postoperative renal failure were observed more frequently in the patients with a previous PCI procedure than in the other group. There was also a significant difference between the 2 groups with regard to the consumption of statins, which was more frequent in the patients with a previous PCI procedure. Given that in many studies conducted so far, the consumption of statins before surgery reduces the mortality and morbidity rates after CABG,^18^-^21^ if such a difference had not existed between our 2 groups, our study might have yielded different results.

Previous studies have shown that renal failure can significantly affect the long-term mortality of patients after PCI, CABG, and other cardiac surgeries.^22^-^24^ In a study by Borner et al.,^6^ renal failure was found more frequently in the PCI group and also a higher mortality risk was reported for the PCI group. In this study, there was a significant difference in the urea level between the PCI and non-PCI groups. We hypothesized that the higher urea level in the PCI group may be an indication for a poor long-term outcome by comparison with the other group.

Our study has certain limitations that should be addressed. Firstly, our study was performed in a single center, which may have caused selection bias. Secondly, this is a cross-sectional study and the long-term outcomes of the patients were not evaluated. Thirdly, a previous history of dual antiplatelet therapy was not accessible to our researcher. Dual antiplatelet therapy is one of the important factors that affect the outcomes after CABG in patients with PCI. Further studies for the long-term follow-up of patients and their comparison based on the number and type of the involved vessels may bolster the selection of the best interventional treatment for patients.

## Conclusion

In this study, PCI was not an independent risk factor for post-CABG mortality; nevertheless, some results such as the postoperative hemorrhage volume, the hospital length of stay, the ICU length of stay, and reduced EFs were worse in the patients with a previous PCI procedure than in those without a history of PCI. This highlights the point that clinicians should be more careful in selecting the method of primary revascularization. We recommend that patients with multiple-vessel disease and patients with the involvement of the proximal left anterior descending artery be directly candidated for CABG because PCI is not suitable in such cases.

## Notes:

***This paper should be cited as: ***Hadadzadeh M, Mirahmadi M, Mirzaei M, Pedarzadeh A, Ghasemzadeh B, Rahimianfar AA. Comparison of Short-term Results between Patients Undergoing Coronary Artery Bypass Graft with a Stent-Placement History and Patients Undergoing Primary Coronary Artery Surgery. J Teh Univ Heart Ctr 2019;14(2):53-58.
